# Progress in Psychiatry

**Published:** 1903-08-08

**Authors:** 


### Medical Progress.

### Progress in Psychiatry.

(*Concluded from page* 313.)

**Pathology of Parasyphilitic Affections, with Special Reference to General Paralysis.**—Professor J. Renaut, of Lyons, in a recent contribution,[11] draws attention to the fact that the reaction of the living tissues to the presence of microbes (*e.g.*, the bacilli of tuberculosis, glanders, and syphilis) produces local changes of varying character. The gumma is not the only neoplasm characteristic of syphilis, and moreover its structure is variable according as it is a gumma of the liver, myocardium, or brain, and these are different from gumma of the skin. A cerebral "gumma" is a nodule of which the periphery consists of proliferated neuroglia, the interior being a mass of degenerated brain-substance the vessels of which have undergone obliterative endarteritis. "Each tissue makes its gumma as it can." The main lesion of constitutional syphilis is arterial. In the cutaneous rash of early syphilis the condition is one of paresis of the circular muscles of the terminal arterioles of the skin very near the surface, thus producing areas of active hyperæmia or redness. A little endarteritis also occurs which is slight and transient in the skin. In later stages the endarteritis becomes more marked, producing at first ischæmia, and subsequently dilatation of the vessels. "Wherever it appears, the specific virus of syphilis produces subacute endarteritis obliterans, causing chronic œdema and new interstitial formation." At first diffuse and affecting vast arterial territories of the skin and body generally, the lesion limits itself later to special organs (central nervous system) or viscera. These later lesions are the so-called parasyphilitic changes. Both the early syphilitic and the later parasyphilitic changes are not in nature distinct from one another. They are, on the contrary, explained as being dependent upon the degree to which the process of syphilitic endarteritis has advanced. This progressive endarteritis sets up a sclerosis of the surrounding tissue, and as the endarteritis progresses so does the sclerosis. In the central nervous system this sclerosis often affects "systems" of nerve-elements or neurons. The central nervous system is attacked late, and the sclerosis and degeneration resulting are confined in the case of tabes dorsalis to the posterior root neurons, and in the case of general paralysis to the cerebral cortex and the association neurons of the "central gray matter" in the pons and bulb. And the vascular and perivascular changes once set up in the brain introduce a circle of morbid conditions which result in grave deterioration of the nutrition and vitality of the nerve-elements affected. The lesions of general paralysis are thus essentially of a syphilitic nature, although they may also be produced by other toxic agencies analogous to but not identical with syphilis.

**Anti-syphilitic Treatment of General Paralysis.** Proceeding on the lines of the anatomical pathology of general paralysis as laid down above by Renaut, Leredde, of Paris, points out[12] that the lesions of general paralysis and of tabes dorsalis may be termed syphilitic, though they do not necessarily conform to the old and incomplete classical description of syphilitic lesions. Nageotte's recent observations show that in tabes the appearance of the lesions is quite consistent with its syphilitic ætiology. This affection is accompanied by lymphocytosis of the cerebro-spinal fluid.[13] By recovery of tabes or general paralysis Leredde means an arrest of the degenerative or destructive process. Fournier in his recent monograph on parasyphilitic affections (1899) quotes nine cases of tabes with arrest of symptoms for 16 years after being under antisyphilitic treatment. Isolated cases have been mentioned by various authors, *e.g.*, Erb, Hammond, and Gowers. Lemoine, of Lille, has published the records of six cases of tabes cured by the "intensive" mercurial treatment, and Leduc, of Paris, has published four others, making a total of 10 cases treated and observed during the years 1886 to 1901. As regards general paralysis of the insane Leredde cites the following cases, viz., six cases published by Lemoine, nine cases of Devay, and three cases under the care of Cassäet, making a total of 18 cases illustrating the highly satisfactory results obtained by the "intensive" mercurial treatment of general paralysis. Of Devay's and Cassäet's cases one each is appended:—(*a*) Devay's case: The patient was a man suffering from general paralysis (1899), with loss of memory, weakness of the lower extremities, disturbance of speech, and of writing, vertigo, and a grandiose and expansive mood. Injection of three-fourths of a grain of calomel weekly was followed by improvement. His memory rapidly returned, speech and writing became normal, and the mental state returned to the normal. At the date of publication (1902) the state of complete recovery was maintained. In the other cases recorded by Devay the treatment included injection of calomel and administration of iodides. (*b*) Cassäet's case: the patient was a male general paralytic with tremor of the lips and hands, pupillary irregularity, disturbances of memory, mental confusion (dementia), and loss of control over the bowel and bladder. Mercurial inunction was followed by improvement, which was rapid at first and slower afterwards. Gradually the mental state became normal, the patient, a merchant, being able to resume his business. For five years further improvement was noticeable, and recovery was considered as complete at the end of that time. Leredde writes, "I have collected from [recent] literature 26 cases which may be considered as showing recovery from general paralysis and from tabes." Anti-syphilitic treatment in paretics and tabetics should therefore begin early. "Delay is dangerous, because while we wait irremediable degenerations may be occurring." For a vigorous adult the full dose is $\frac{1}{2}$ to $\frac{3}{4}$ grain of mercuric benzoate in normal salt solution, or an equal quantity of mercuric biniodide in a dilute solution of potassium iodide. The injection should begin with a $\frac{1}{2}$-grain daily dose, gradually increased until the full dose ($\frac{3}{4}$ grain) is given. Meanwhile symptoms of "intolerance" should be carefully watched for. If indigestion, loss of flesh, and febrile reaction occur the limit has been overstepped and the dose should be reduced. The mouth of the patient should be kept scrupulously clean. The dangers of "intensive mercurial treatment" are in this way minimised. By very gradually increasing the daily dose is general paralysis, we may, says Leredde, prevent any sudden action of the mercury and thus avoid therapeutic accidents.

[11] Philadelphia Med. Jour., Jan. 17, 1903. [12] Ibid., April 18, 1903. [13] La Presse Médicale, Dec. 10, 1902.

